# Prospective clinical evaluation of chairside-fabricated zirconia-reinforced lithium silicate ceramic partial crowns—5-year results

**DOI:** 10.1007/s00784-021-04132-y

**Published:** 2021-08-20

**Authors:** Sven Rinke, Tanja Zuck, Tim Hausdörfer, Andreas Leha, Torsten Wassmann, Dirk Ziebolz

**Affiliations:** 1 Department of Prosthodontics, University Medical Center, Robert-Koch-Str. 40, 37075 Goettingen, Germany; 2 Department of Preventive Dentistry, Periodontology, and Cariology, University Medical Center, Goettingen, Germany; 3 Department of Medical Statistics, University Medical Center, Goettingen, Germany; 4 Department of Cariology, Endodontology and Periodontology, University Medical Center, Leipzig, Germany

**Keywords:** High-strength glass–ceramics, Partial crown, Survival rate, Success rate, Clinical study

## Abstract

**Objectives:**

A university-based randomized clinical study evaluated the 5-year performance of chairside-fabricated zirconia-reinforced lithium silicate (ZLS)-ceramic partial crowns.

**Material and methods:**

Forty-five patients were restored with 61 chairside-fabricated ZLS-restorations (Cerec SW 4.2, Dentsply Sirona, Germany; Vita Suprinity, Vita Zahnfabrik, Germany). Deviating from the manufacturers’ recommendations, restorations with reduced minimum material thicknesses (MMT) were fabricated: group 1, MMT = 0.5–0.74 mm (*n* = 31); group 2, MMT = 0.75–1.0 mm (*n* = 30). For luting, a self-adhesive cement (SAC) or a total-etch technique with a composite cement (TEC) was applied. Statistical evaluation was performed by time-to-event analysis (Kaplan–Meier). Possible covariates of the survival (SVR) and success rates (SCR), evaluated in a Cox regression model, were MMT, restoration position (premolar/molar), and cementation technique (SAC vs. TEC).

**Results:**

Forty patients (54 restorations, premolars, *n* = 23; molars, *n* = 31) participated in the 5-year follow-up. Five losses due to ceramic fractures occurred in group 1 (*n* = 28) (SVR: 83.0% [95% confidence interval (CI): 0.71–0.96]). Group 2 (*n* = 26) showed no losses (SVR: 100%). The success rate for partial crowns placed on premolars was 100% and 69% (95% CI: 0.54–0.84) for molar restorations. Recementation was required in 4 restorations with SAC (SCR: 86% [95% CI: 0.73–0.99]; SCR-DC: 100%). Restorations in group 2 showed a significantly reduced risk of material fracture hazard ratio (HR) = 0.09, *p* = 0.0292) compared with the restorations in group 1. Molar partial crowns showed an increased risk for a clinical intervention (HR = 5.26, *p* = 0.0222) compared to premolar restorations.

**Conclusions:**

Material thickness and position of the restoration are risk factors influencing the survival and success rate of ZLS-ceramic partial crowns.

**Clinical relevance:**

Observation of an MMT of at least 0.75–1.0 mm for ZLS-ceramics is essential to avoid material-related fractures.

**Clinical trial registration**: German Clinical Trails Register (trial number: DRKS00005611)

## Introduction

In patients with extensively destroyed natural teeth, a partial crown restoration can be a minimally invasive alternative to full crowns [1, 2]. Apart from resin-based materials, dental ceramics are the group of materials with the longest clinical history of use for the fabrication of tooth-colored partial crowns [3, 4]. The available long-term clinical documentation indicates that up to now, a material fracture is the most frequent cause of the loss of all-ceramic partial crowns, even those fabricated according to the manufacturer’s recommendation with a minimum material thickness (MMT) of 1.5 mm [5–8]. This observation is based on the fact that in these studies, mostly feldspathic or leucite-reinforced glass–ceramics with a mean flexural and tensile strength of less than 200 MPa were used [5, 7]. Thus, dental ceramic materials with improved mechanical properties offer the possibility of reducing the incidence of material-related failure [9].

High-strength glass–ceramics, such as lithium disilicate ceramics or newly developed zirconia-reinforced lithium silicate (ZLS) ceramics with a flexural and tensile strength of 370–450 MPa, provide stability 2- to 3-times greater than that of leucite-reinforced glass–ceramics [10–13]. Clinical studies with mean observational periods of 11 years have demonstrated that the improved mechanical properties of lithium disilicate materials led to significantly reduced material-related failure rates of ceramic onlays and partial crowns [14–16]. Nevertheless, for the more recently introduced ZLS-ceramics, the evidence for a reduced fracture rate is still limited as of now because only studies with an observational period of up to 3 years are available [17–19]. Moreover, several in vitro studies have evaluated the effect of the MMT on the fracture characteristics of indirect all-ceramic restorations. These evaluations focused on determining whether the improved mechanical properties of the new high-strength ceramic materials allowed a reduction in the MMT [20–23]. Based on these results, several manufacturers have reduced the recommended material thickness for high-strength glass–ceramic restorations to 1 mm.

However, it must be considered that the reasons for the failure of all-ceramic restorations are multifactorial. Apart from the material, fabrication technique, and type of cementation, patient-related factors, such as bruxism or location of the restoration, have had clinically relevant effects on the long-term durability of all-ceramic restorations [24–26].

A general recommendation for a reduced MMT for all-ceramic restorations made of high-strength glass–ceramics should therefore be based on clinical studies. These studies are still sparse. Therefore, clinical data from additional studies are mandatory to generate an evidence-based recommendation [18, 27].

The present prospective clinical study aimed to evaluate the risk factors for the material-induced failure of chairside-produced (Cerec system, Dentsply Sirona, Bensheim, Germany) ZLS-ceramic partial crowns (Vita Suprinity, Vita Zahnfabrik, Bad Säckingen, Germany) with a special consideration of the applied cementation technique and a reduced MMT of 0.5 to 1.0 mm. The following null hypothesis was formulated: The survival and success rates are independent of the cementation technique applied and the MMT of the restorations.

## Materials and methods

### Patient recruitment

Forty-five adult patients (28 female/17 male) with indications for a chairside-fabricated partial crown (covering all cusps) were included according to the following inclusion criteria:

- vital and symptom-free posterior teeth (premolar/molar)
- existing antagonistic teeth
- at least one proximal contact.

Patients fulfilling the following criteria were excluded:

- Clinical symptoms of bruxism
- Preparation unsuitable for optical impression taking (e.g., deeply subgingival)
- Nonvital or endodontically treated teeth
- Untreated periodontal disease
- Age less than 18 years

A maximum of two partial crowns per patient was allowed. The study protocol was evaluated and approved by the ethics committee of the medical faculty of Georg-August University, Goettingen, Germany (No. 27/7/13). All patients participating in the study provided written informed consent.

### Treatment

The clinical treatments and the manufacturing of the ceramic restorations were performed during a 6-month period (January-July 2014) by two experienced dentists in the Department of Preventive Dentistry, Periodontology, and Cariology, University Medical Center, Goettingen, Germany. All patients received professional prophylaxis including instructions for oral homecare procedures before the treatment. The preparation of the teeth followed the published recommendations for all-ceramic partial-crown restorations [1, 4, 28].

After preparation, a powder-free intraoral scanning device (Cerec AC Omnicam, Dentsply Sirona, Bensheim, Germany) was used for optical impression taking. The monolithic partial crown restorations (Vita Suprinity, Vita Zahnfabrik, Bad Säckingen, Germany) were constructed with CAD software (Cerec software 4.2, Dentsply Sirona, Bensheim, Germany). They were fabricated in a wet-milling unit (Cerec MCXL, Dentsply Sirona, Bensheim, Germany). Subsequently, the restorations were crystallized and stained according to the manufacturer’s instructions. After the try-in of the restoration, the intaglio surfaces of the restorations were conditioned with hydrofluoric acid (5%, Vita Ceramics Etch, Vita Zahnfabrik, Bad Säckingen, Germany) for 20 s and then rinsed with water and dried with water-free and oil free air. Finally, all restorations were silanized (Monobond S, Ivoclar Vivadent, Schaan, Liechtenstein, exposure time: 1 min).

The adhesive luting of the partial crowns was performed at random, either using a dual-curing composite cement (TEC; *n* = 30) and multibottle bonding with the total-etch technique (Syntac classic and Variolink II, Ivoclar Vivadent, Schaan, Liechtenstein) or with a self-adhesive cement (SAC, RelyX Unicem, 3 M, Deutschland GmbH, Neuss, Germany; *n* = 31), each with rubber dam application. In the SAC group, the prepared tooth was cleaned with a slurry of pumice, rinsed with water spray, dried shortly avoiding overdrying, and the luting agent was applied directly into the cavity without any further pretreatment of the hard tooth tissues. In the TEC group, the prepared tooth surfaces were treated with the total-etch technique using 37% phosphoric acid gel condition the hard tissues (dentin 15 s/enamel 30 s). The bonding agent (Syntac classic) was applied after the acid was rinsed off, and the preparations were carefully air-dried. The luting agent (Variolink II) was applied directly to the cavity. For the random assignment of participants to the cementation groups, an online statistical computing web program (www.randomization.com) was used to generate the randomization schedule.

After removing the excess cement, the luting agent of each restoration was polymerized for 120 s. Then, occlusion was restored according to the initial situation (anterior-canine guided occlusion or unilateral dynamic guidance).

**Fig. 1:**
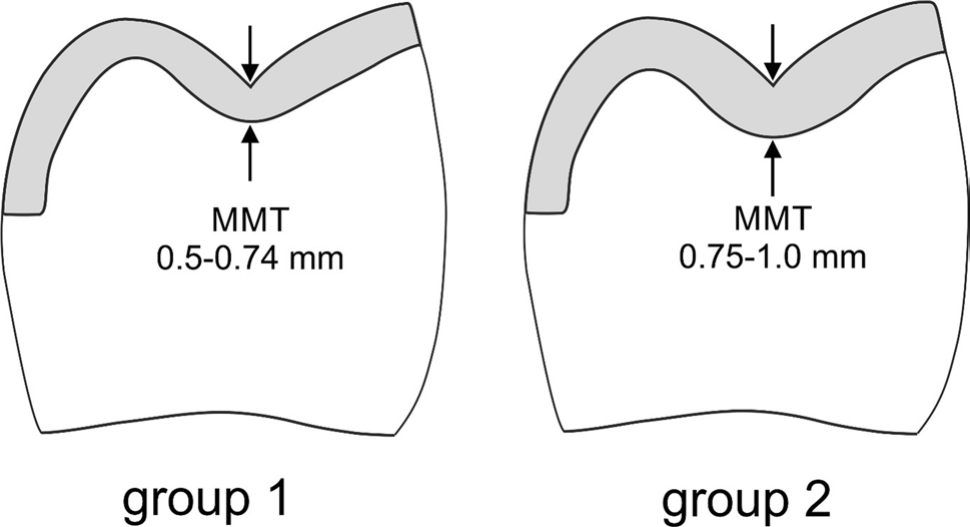
Schematic of the preparation design and group distribution related to the occlusal minimum material thickness (MMT). Group 1: MMT = 0.5–0.74 mm. Group 2: MMT = 0.75–1.0 mm

However, because the standard setting for the MMT of the construction software (Cerec software 4.2) at the time of the clinical treatment phase was 0.7 mm, all restorations were accidentally produced with a reduced MTT, thus violating the manufacturer’s recommendations for the MMT being 1.0 mm. This deviation from the original treatment protocol was only realized after the clinical treatment phase had been completed. The ethics committee was informed about this deviation from the study protocol and approved the continuation of the study with a modified study protocol, including the reduced MMT as a risk factor for the survival and success of the restorations. For this purpose, the generated construction data were analyzed regarding the MMT. Bucco-lingual cross sections of the construction data were analyzed with the measuring function of the Cerec SW. The minimum material thickness for all restorations occurred in the central fissure; therefore, this area was used to divide the fabricated ceramic partial crowns into two groups (Fig. 1).

-
  - Group 1: MMT in the central fissure of 0.5–0.74 mm
-
  - Group 2: MMT in the central fissure of 0.75–1.0 mm

### Clinical evaluation

The restorations were evaluated at the time of cementation (baseline) and followed by clinical examinations every 12 months using modified United States Public Health Service (USPHS) criteria that have been used in other clinical studies evaluating the clinical performance of ceramic partial crowns [29–31].

**Fig. 2:**
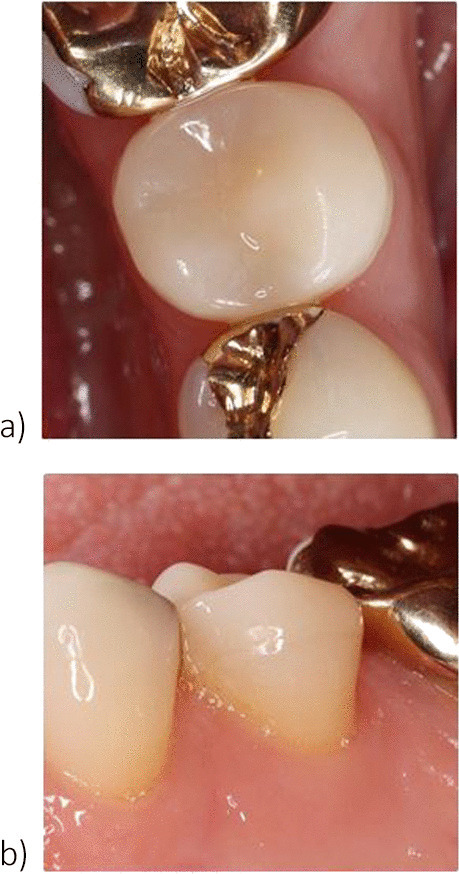
**a**, **b** Clinical situation of an adhesively luted (TEC group) second lower premolar at the 5-year clinical evaluation: **a**. Occlusal view, **b**. buccal view. The restoration was rated “alpha” for the USPHS criteria marginal adaptation and marginal discoloration

During the annual follow-up examinations, all restorations were clinically evaluated with a mirror, a number 9 dental probe (22,243.20; Stoma Dentalsysteme GmbH, Emmingen-Liptingen, Germany), and intraoral digital photographs. The vitality of the teeth was confirmed by CO_2_ testing. Every restoration was examined regarding fissures, fractures, loosening, caries; the following modified USPHS criteria were used to rate the marginal adaptation and marginal discoloration [30, 31] (Fig. 2a,b):

**Table:** 

Marginal adaptation	alpha	Margin not discernible, probe does not catch
	bravo	Probe catches on margin but no gap; dentin or liner exposed
	charlie	Probe catches on margin and gap on probing, dentin or liner exposed
	delta	Restoration fractured or missing
Marginal discoloration	alpha	No marginal discoloration
	bravo	Marginal discoloration, not penetrated toward pulp
	charlie	Marginal discoloration penetrated toward pulp

Failures that occurred before the examinations due to negative events (restoration loss, recementation if necessary, ceramic fracture, biological complications) were documented in the patient files and considered in the final results [18, 19].

The last follow-up examinations were performed between January and May 2019 by a trained dentist (T.P.) who was not involved in the placement. The training regarding the survival and success criteria [32] was performed by one of the authors (D.Z.) and repeated until each examiner had a substantial correlation, as measured by Cohen’s Kappa (*k* ≥ 0.6).

### Statistical analysis

The statistical analysis was based on information on the survival and success rates of the reconstructions. Survival was defined as the restoration being in situ at the time of the follow-up examination without signs of a total loss (i.e., the in situ criterion) [32]. A total loss was defined as a clinically unacceptable ceramic fracture of the restoration or a biological event (caries, tooth fracture, or periodontal disease) requiring complete replacement of the restoration or removal of the affected tooth. Success was defined as the reconstruction remaining unchanged and functional in situ without any intervention throughout the total observational period [32]. The survival time of a restoration was defined as the period between baseline (day of cementation) and either the last follow-up examination or, in the case of failure, the day of failure documentation in the patient file. The time-dependent survival rates of the restorations (based on the in situ criterion) and the success rates (intervention-free) of the partial crowns were calculated by Kaplan–Meier survival analysis.

The MMT (0.5–0.74 mm vs. 0.75–1.0 mm), the position of the restoration (premolar vs. molar), and the cementation technique (SAC vs. TEC) were evaluated as possible covariates of the time-dependent survival and success rates.

Different observations in the same patient (several partial crowns per patient) were evaluated as dependent based on the adapted estimation of variance in the Cox regression model. Therefore, a marginal model was applied for data analysis [33]. Univariate Cox regression was performed for every influencing factor. Penalized Firth correction was employed in the model in case of complete data separation. A *p* value of less than 0.05 was accepted as statistically significant. Statistical analyses were performed with the statistical software R (version 3.5.3) using the R package “survival” (version 2.44.1.1) and the “prodim” module for the time-to-event analyses. The changes in the clinical criteria over time between baseline and the 5-year examination were evaluated separately for each luting procedure using the chi-square test (*α* = 0.05).

A post hoc sample size calculation based on the incidence of prosthesis failures was conducted to determine the number of participants needed per group to confirm a statistically significant difference between the treatments with *α* = 0.05 and a power of 80%.

## Results

### Study population

From January to June 2014, 45 patients were included in the present study and restored with a total of 61 partial crowns. One female patient withdrew her written consent for participation in the study at the time of the baseline examination, and these data were excluded. Four patients (1 female/3 male) with 5 molar restorations were lost during the follow-up period or declined further participation in the study. Two patients relocated from the area, one patient was unable to attend the last clinical follow-up due to severe illness, and one patient died. Their data were censored at the date of the last clinical evaluation or notification of a failure/intervention. Forty patients (26 female/14 male, recall rate: 90.9%) with a total of 54 partial crowns participated in the 5-year follow-up examination. This clinical examination was carried out between January and May 2019 (mean observational period: 56 ± 10 months). Twenty-eight restorations were allocated to group 1, and the remaining 26 restorations were assigned to group 2. From the finally examined restorations, thirty-one were placed in the maxilla, and 23 were in the mandible (23 premolar and 31 molar partial crowns). Twenty-five of the examined restorations were luted adhesively by using the total-etch technique (TEC), and 29 partial crowns were luted with an SAC material.

### Survival rate

**Fig. 3:**
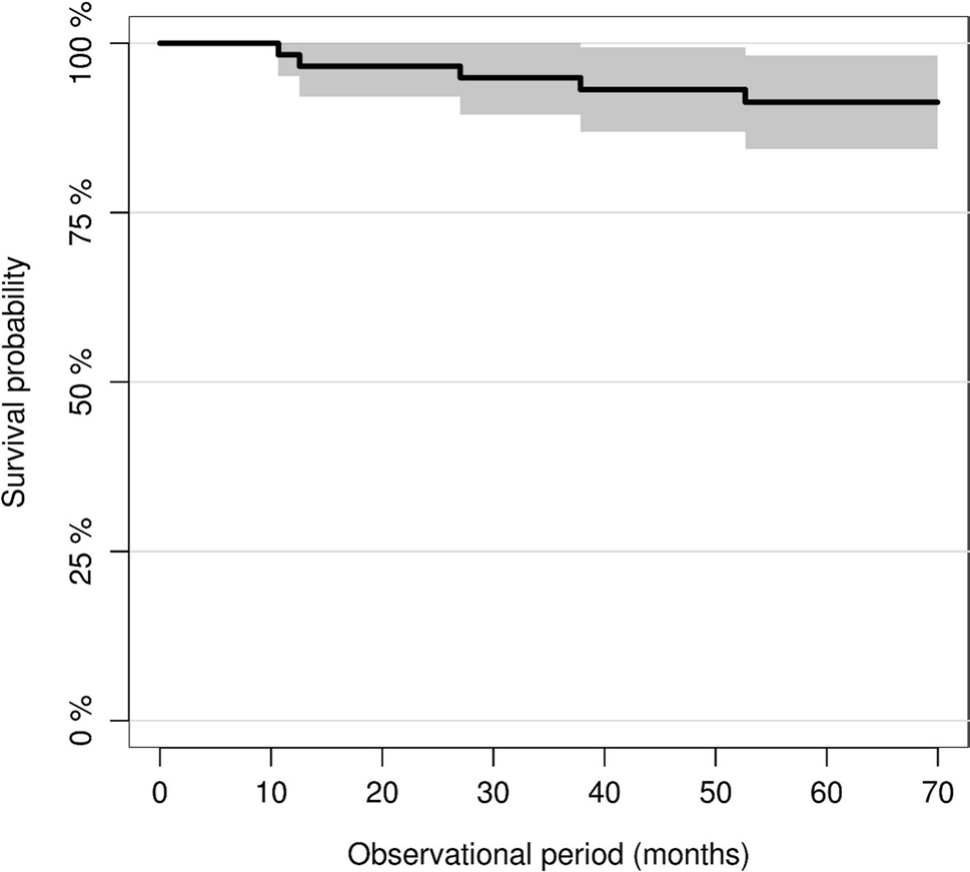
Overall survival rate of the chairside-fabricated zirconia-reinforced lithium silicate (ZLS) partial crowns after a mean observational period of 5 years

Five restorations failed completely during the 5-year follow-up period. The overall survival rate after 5 years was 91% (95% confidence interval (CI): 0.84–0.98) (Fig. 3). All complete failures occurred in group 1 (MMT = 0.5–0.74 mm), and they were related to catastrophic material fractures involving the area where the MMT was measured (i.e., central fissure). Furthermore, all complete failures occurred for restorations placed on molars. The time-dependent 5-year survival rate was 83% (95% CI: 0.71–0.96) in group 1 and 100% in group 2 (MMT = 0.75–1.0 mm).

**Fig. 4:**
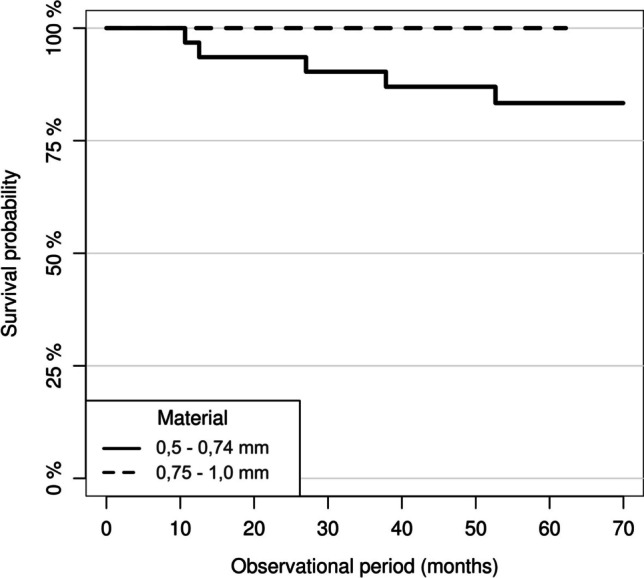
Survival rate of the partial crown restorations by the occlusal minimum material thickness (MMT)

**Fig. 5:**
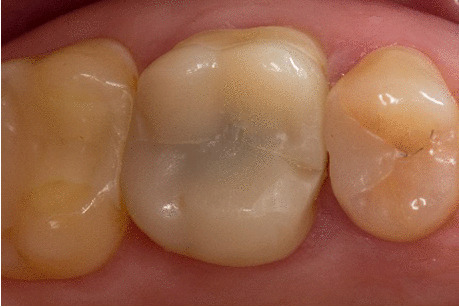
Fracture of an ZLS partial crown from group A (MMT 0.5–0.74 mm). The fragments of the restoration were mobile at the date of the clinical examination

With a hazard ratio (HR) of 0.09, the Cox regression analysis showed an 11.1-fold reduced risk of material fracture in restorations with an MMT of 0.75–1.0 mm compared to restorations with an MMT of 0.5–0.74 mm. This effect proved to be statistically significant (*p* = 0.029) (Figs. 4 and 5). The 5-year survival rate for ZLS partial crowns placed on molars was 85% (95% CI: 0.74–0.96) and 100% for premolar restorations. The univariate Cox regression model revealed a 7.85-fold increased risk for a complete failure for molar restorations compared to premolar restoration. However, this effect proved not to be significant (0.0673). The survival rate of the partial crowns inserted with TEC and the total-etch technique was 93% (95% CI: 0.85–1), while the partial crowns inserted with SAC showed a survival rate of 90% (95% CI: 0.8–1). The cementation technique (SAC vs. TEC, HR = 1.43, *p* = 0.677) showed no significant influence on the survival rate of the partial crowns.

Therefore, the null hypothesis has to be partly rejected regarding the survival rate, as a significant effect of the MMT could be demonstrated, while no effect of the cementation mode or the position of the restoration was detected.

### Success rate

**Fig. 6:**
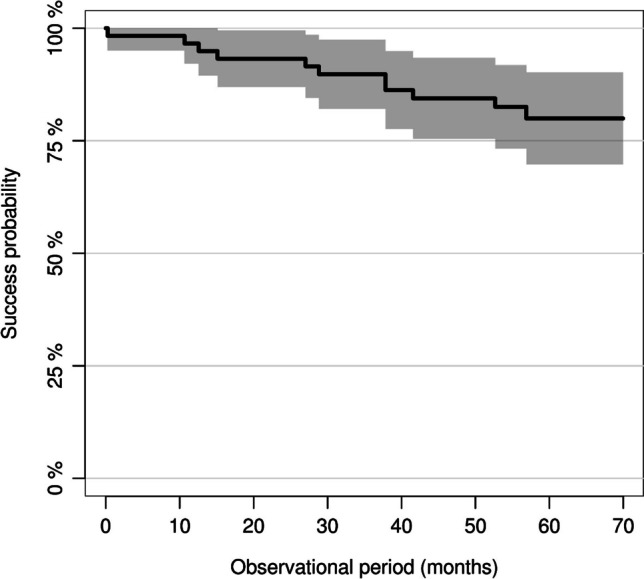
Overall success rate of the chairside-fabricated zirconia-reinforced lithium silicate (ZLS) partial crowns over a mean observational period of 5 years

Apart from 5 total losses, six clinical interventions for six different restorations (6 patients) were necessary to maintain function. The time-dependent overall 5-year success rate (intervention-free survival) was 80.0% (95% CI: 0.7–0.9) (Fig. 6). Four clinical interventions necessary to keep the restorations functional were caused by the loss of the retention of partial crowns inserted with SAC. The 4 restorations did not show any defects and were recemented with the same material. One additional intervention was caused by minor ceramic fractures < 2 mm^2^ (polishing) and the loss of pulp vitality (endodontic treatment). Further interventions due to temperature sensitivity, loss of vitality, or secondary caries were not necessary (100% alpha ratings for the USHPS criteria “postoperative sensitivity”, and “recurrent caries”). The specific success rate of restorations in group 2 (MMT = 0.75–1.0 mm) luted with the TEC material was 100%.

**Fig. 7:**
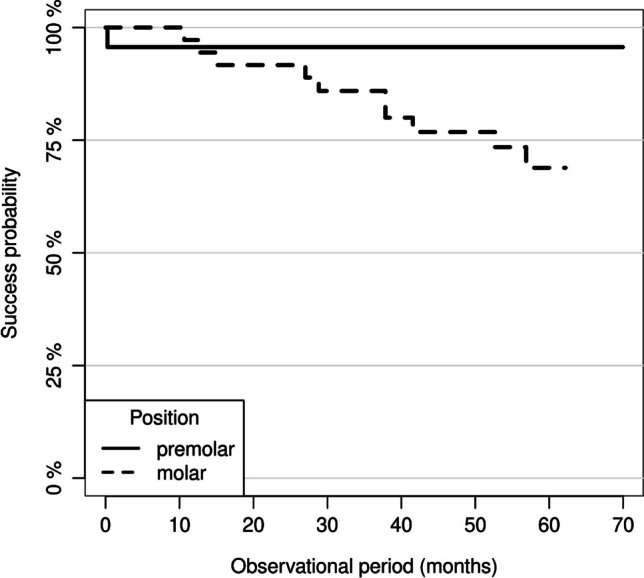
Success probability of the partial crown restorations according to the tooth position

Based on the Cox regression model, the overall success rate (intervention-free survival) was independent of the MMT (0.5–0.74 mm vs. 0.75–1 mm, *p* = 0.844), and the cementation technique (*p* = 0.124). The success rate for partial crowns placed on premolars was 100% and 69% (95% CI: 0.54–0.84) for molar restorations. The univariate Cox regression revealed a 5.26-fold increased risk for partial crowns placed on molars for a failure or a clinical intervention compared to premolar restoration. This effect proved to be statistically significant (*p* = 0.0222) (Fig. 7). The null hypothesis was partly rejected regarding the success rate.

**Fig. 8:**
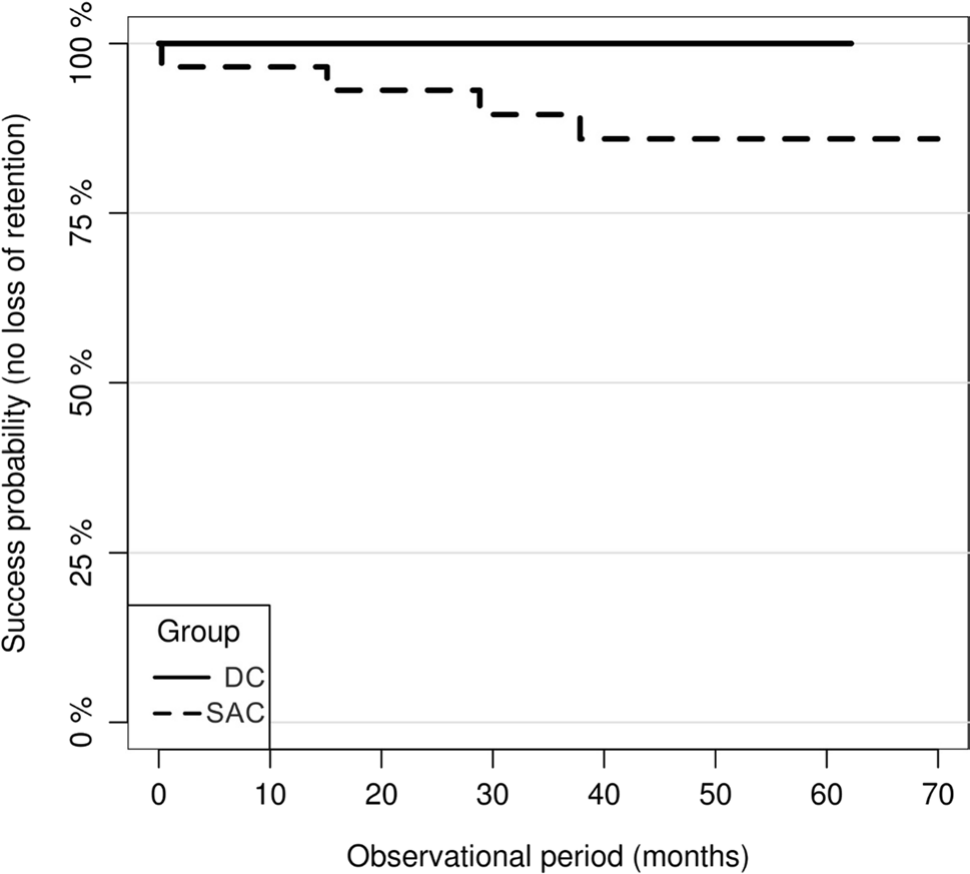
Success probability of the partial crown restorations based on the loss of retention according to the cementation technique. TEC: total-etch technique with dual-curing cement, SAC: self-adhesive cement

The specific 5-year success rate, referring to the event “loss of retention,” was 86% (95% CI: 0.73–0.99) for partial crowns inserted with SAC, whereas the success rate (no loss of retention) for the TEC-fixed restorations was 100% (Fig. 8).

Related to the event loss of retention, partial crowns that were cemented with SAC showed a 9.2-fold greater risk of requiring recementation than restorations cemented with TEC. Although this association was not statistically significant (*p* = 0.0527), it shows a strong tendency toward an influence of the cementation technique on the success rate of partial crowns.

At baseline, the marginal adaptation was rated alpha in 29 restorations (96.7%) in group TEC and 27 restorations (93.1%) in group SAC. All other restorations were rated bravo. At the 5-year recall, the alpha ratings decreased to 36.0% in group TEC and to 23.1% in group SAC (Table 1).

**Table 1:** Ratings for selected USPHS criteria at baseline, 2-year, and 5-year clinical examination

		Marginal adaptation^4^				Marginal discoloration^4^		
Group	Time	alpha	bravo	charlie	delta	alpha	bravo	charlie
TEC	Baseline (*n* = 30)	29 (96.7%)	1 (3.3%)	0	0	30 (100%)	0	0
	2 years (*n* = 28)^1^	23 (82.1%)	5 (17.9%)	0	0	21 (75.0%)	7 (25.0%)	0
	5 years (*n* = 25)^2^	9 (36.0%)	15 (60.0%)	1 (4.0%)	0	14 (56.0%)	6 (24.0%)	5 (20.0%)
SAC	Baseline (*n* = 29)	27 (93.1%)	2 (6.9%)	0	0	29 (100%)	0	0
	2 years (n = 29)	21 (72.4%)	8 (27.6%)	0	0	20 (69.0%)	9 (31.0%)	0
	5 years (*n* = 26)^3^	6 (23.1%)	18 (69.2%)	2 (7.7%)	0	12 (46.2%)	7 (26.9%)	7 (26.9%)

^1^Only 28 restorations remained because two partial ceramic crowns failed prior to the follow-up examination

^2^Only 25 restorations remained because three patients did not attend follow-up examinations

^3^Only 26 restorations remained because three partial ceramic crowns failed prior to the follow-up examination

^4^Significant difference between baseline and 2-year and 5-year investigation (*p* ≤ 0.05)

At baseline, the marginal discoloration was rated alpha for all restorations in both groups. Until the 5-year clinical examination, the alpha ratings decreased to 56.0% (group TEC), resp. 46.2% (group SAC). Regarding marginal adaptation and marginal discoloration, a statistically significant difference (*p* < 0.05) could be found in both groups for the data assessed at baseline and after 5 years (Table 1).

Regarding the parameters “marginal adaptation” and “marginal discoloration,” no significant differences for the data assessed at the 5-year recall were detected between the restorations in group TEC and group SAC.

## Discussion

After a mean observational period of 5 years, in the present study, a significant effect of the MMT of chairside-fabricated ZLS-ceramic partial crowns on the survival rate could be detected. A survival rate of 100% at an MMT of 0.75–1.0 mm was determined, while the 5-year survival rate of partial crowns with an MMT of < 0.75 mm was significantly reduced to 83% (95% CI: 0.71–0.96). This effect has not been documented in clinical trials yet. Apart from the MMT, the position of the restorations (premolar vs. molar) had a significant effect on the 5-year success rate of chair-side fabricated ZLS partial crowns. Restorations luted with SAC showed a pronounced tendency for an increased risk for a loss of retention (HR: 9.2). These findings are in good accordance with the results of other clinical studies on ceramic partial crowns [5–8, 29–31].

For the interpretation of the results of the present study, it should be considered that the findings of the clinical investigation are influenced by a variety of variables, e.g., study design (prospective vs. retrospective, fabrication technique, evaluation criteria, observational period). Therefore, the results of the present study should preferably be compared with those of clinical studies on chairside-fabricated all-ceramic partial crowns with a similar design (prospective), using comparable survival and success criteria, and reporting a comparable observational period [18, 19, 29–31, 34–36].

Federlin et al. (2010) documented a survival rate of 88.8% after 5.5 years for chairside-fabricated feldspathic ceramic partial crowns (Vita MK II, Vita Zahnfabrik, Bad Säckingen, Germany) with an MMT of 1.5 mm. Material fracture was the most frequent cause of failure [30]. This is in good accordance with the findings of another prospective clinical trial evaluating the clinical performance of chairside-fabricated feldspathic ceramic partial crowns luted with an SAC. At the 3-year recall, 7 out of 12 complete failures were attributed to catastrophic material fractures [31].

For chairside-fabricated ceramic partial crowns fabricated from a leucite-reinforced ceramic material (ProCAD, Ivoclar Vivadent, Schaan, Liechtenstein), a 3-year survival rate of 97% was reported [34]. From another prospective clinical trial on chairside-fabricated leucite-reinforced glass–ceramic onlays (Empress CAD, Ivoclar Vivadent, Schaan, Liechtenstein), a material fracture rate of 6.7% during a 5-year observational period was reported [36]. In both studies, the restorations were fabricated with a minimum material thickness of 1.2–1.5 mm according to the manufacturers’ recommendations. Interestingly, an additional study on conventionally fabricated partial crowns made from the same glass–ceramic material reported that a mere reduction in the MMT to 1.0–1.4 mm caused a significant increase in fracture-related loss after a mean observational period of 3 years [37].

To date, a limited number of clinical trials with maximum observational periods of 3 years have evaluated chairside-fabricated ZLS-ceramic partial crowns [17–19]. From a practice-based study evaluating chairside-fabricated ZLS-ceramic partial crowns (Celtra Duo, Dentsply Sirona, Bensheim Germany), a 3-year survival rate of 99% (CI-95%: 0.97–1) was reported. The restorations were adhesively luted with TEC materials and fabricated with an MMT of 1.5 mm. Furthermore, 2-year results from the present study have already been published. During this follow-up, 2 material fractures required the replacement of 2 restorations from group 1 (2-year survival rate group 1: 94% CI-95%: 0.85–1)). No losses occurred in group 2 (2-year survival rate group 2: 100%) [18].

For the 5-year follow-up reported in the present study, no fracture-related losses were documented with a moderate reduction relative to the manufacturer-recommended MMT (0.75–1.0 mm), independent of the applied cementation technique. Thus, the survival rate was in the same range as that of chairside-fabricated partial crowns made of other ZLS-ceramic materials and conventionally fabricated partial crowns made of lithium disilicate ceramic materials with an MMT of 1.5 mm [19, 34]. Fracture-related losses occurred only after a massive reduction relative to the recommended MMT (0.5–0.74 mm).

In this group, the survival rate after a mean observational period of 5 years was 83% due to 5 fractures. Statistical analysis showed that the survival rate was dependent on the MMT of the partial crowns (MMT of 0.5–0.74 mm vs. 0.75–1.0 mm, *p* = 0.0292). The evaluated HR of 0.09 showed an 11.1-fold reduced risk of material fracture, demonstrating a significant influence of the MMT on the fracture-related failure of chairside-fabricated partial crowns made of a ZLS-ceramic material.

It was taken into account that due to necessary occlusal adjustments the finally cemented restoration could be thinner than the dimensions measured from the construction data. Nevertheless, in the course of the present study, only minor adjustments of the cemented restoration were carried out with fine grit size diamond instruments and were mainly related to the cusp areas. Therefore, this seems to be a low risk for a bias of the results.

The effect of the MMT on the fracture strength of ceramic crowns and partial crowns has been evaluated only in several in vitro studies [20–23] and a limited number of clinical studies [18, 27]. Based on these investigations, it was postulated that for high-strength glass–ceramic materials (e.g., lithium-disilicate ceramics or ZLS-ceramics), the MMT can be reduced to 1.0 mm.

Considering the limitations, the results of the present study can be interpreted as an initial clinical verification that for high-strength glass–ceramic materials, the occlusal MMT can be reduced to 1.0 mm without an increase in the rate of fracture-related failures. This represents a new finding not reported from a clinical trial yet. However, to specify a defined limit for an MMT leading to fracture-related failures, additional studies with larger populations and longer observational periods are required [29, 32].

Although all complete failures in the present study occurred exclusively on molars, the statistical analysis failed to determine a statistically significant effect of the position of the restoration on the survival rate. This might be related to a small subgroup with a skew distribution of the restorations (23 premolar restorations/36 molar restorations) and a limited number of observations. These aspects are indicators that the study was underpowered. Therefore, a post hoc power analysis was performed for the risk factor “position of the restoration.” Post hoc sample size calculation based on the incidence of prosthesis failures revealed that 50 restorations would be needed per group to confirm a statistically significant difference between the molar and premolar restorations 5 years after prosthesis insertion.

Clinical interventions for maintaining the function of the restorations during the 5-year observational period were mainly required due to a loss of retention. Endodontic treatment was needed for one out of 54 restorations (complication rate 1.9%) during a 5-year functional period. No secondary caries were observed. These findings are in good accordance with other clinical studies reporting on the clinical performance of adhesively luted ceramic partial crowns [18, 29–31].

A loss of retention (*n* = 4) occurred only in restorations that were luted with SAC, while restorations inserted with TEC showed no loss of retention. Statistical analysis revealed a pronounced tendency toward an increased risk (HR = 9.2) of retention loss in restorations inserted with SAC. However, this effect was not statistically significant (*p* = 0.0527). Post hoc sample size calculation based on the incidence of loss of retention revealed that the sample size of the subgroups was too small to confirm a statistically significant difference between the two cementation techniques.

Nevertheless this observation is in good accordance with the results of other clinical studies showing increased rates of retention loss in chairside-fabricated partial crowns inserted with SAC [29, 31, 35].

A possible explanation of the increased debonding rate can be seen in the fact that the bond strength of self-adhesive resin cements to the tooth surface (enamel and dentin) was reported to be inferior to conventional composite resin cements [38–40]. This fact is of importance as in the present clinical study, a non-retentive preparation design for the partial crowns was used. For these preparations where dentin was predominant, cements with higher bond strength as total-etch or self-etch resin cements may provide a better protection against a loss of retention [29, 35].

In the present study, all restorations could be recemented after the loss of retention. In contrast, in clinical studies on chair-side fabricated restorations made from feldspathic materials, the loss of retention was mostly related to restoration fracture, so no recementation was possible [29, 31, 35]. This observation can be explained by the increased mean flexural strength of the applied ZLS-ceramic materials in comparison with feldspathic ceramic materials [10–13].

Regarding the success rate in the present study a significant influence of the tooth position (premolar vs. molar) could be detected. Restorations placed on a molar revealed a 5.26-fold increased risk for a failure or a clinical intervention to maintain function. This is in good accordance with the findings of other clinical trials and can be attributed to higher occlusal load in the molar area compared to the premolar area [5–8].

With respect to clinical changes (USPHS criteria) over time, statistically significant differences were determined between baseline and the 5-year recall regarding the criteria for marginal adaptation and marginal discoloration. Both criteria showed a statistically significant increase in bravo ratings over time for both cementation techniques, along with a statistically significant decrease in alpha ratings (*p* ≤ 0.05). This is in accordance with the findings of previous studies reporting USPHS criteria changes in the same range for CAD/CAM-fabricated partial crowns from different materials [18, 29–31].

An explanation for this observation can be seen in the wear of the luting material and an increased width of the luting space of the CAD/CAM-fabricated partial crowns [29–31]. Furthermore, for the SAC used in the present study, wear of the resin matrix and loss of fillers resulting in an increase in roughness were reported as reasons for increased staining capacity over time [31]. Additionally, individual patient parameters (diet, smoking habits, and oral home care procedures) must be taken into account [30, 31].

The results of the post hoc power analysis indicated that the study was—due to the small study population and limited number of events—underpowered. Therefore, possible effects of some of the evaluated variables on the survival and success rates could not be verified by the statistical analysis.

This represents a major limitation of the present study. In particular, the importance of a prolonged observational period to detect possible risk factors influencing the clinical performance can be seen when the results of the present study are compared with the previous results reported from the same study population [18]. After a mean observational period of 2 years, two complete failures were reported. The number of failures increased during the following 3 years to 5 failures. Due to the limited number of events, a risk analysis based on the 2-year results was not possible. With the prolonged observational period, the number of events increased and allowed for a sound statistical analysis. Thus, the abovementioned limitations of the studies could be partially compensated by the prolonged observational period. The importance of prolonged observational periods (≥ 5 years) has been described in other prospective studies as well [29, 31].

Another limitation is the deviation from the original study protocol caused by an operating error during the construction process that led to a violation of the MMT (1.0 mm) recommended by the manufacturer. Thus, the restorations were not randomized regarding this parameter. This is a limitation for a clinical research project. On the other hand, this inadvertent deviation from the study protocol provided important evidence on the possible clinical impact of errors in the fabrication process of chairside-fabricated restorations. To the best of the authors’ knowledge, this impact has not yet been documented in the literature.

Despite these limitations, the present study provides important information on the clinical safety and performance of chairside-fabricated partial crowns because it offers clinical data for relatively new ZLS-ceramic materials, which have been evaluated in a limited number of clinical studies with shorter observational periods (≤ 3 years) only [17–19]. Moreover, this study provides the first clinical data on chairside-fabricated partial crowns with a reduced MMT, generating important information to verify the manufacturers’ recommendations based on in vitro data only for a less invasive preparation design for high-strength glass–ceramic restoration (MMT: 1.0 mm). Furthermore, the study verifies the relevance of the cementation technique and restoration position for the long-term evaluation of all-ceramic partial crowns, as already documented in other clinical studies [5–8, 29, 31, 35].

However, due to the limited sample size, a general clinical recommendation must be made with caution, as the present study was underpowered to detect significant effects of potential risk factors other than the MMT and tooth position. Therefore, further clinical studies with longer observational periods and larger sample sizes are needed.

## Conclusions

Chairside-fabricated ZLS-ceramic partial crowns showed a 5-year survival rate of 100% with a moderate reduction relative to the manufacturer’s recommendations regarding the occlusal MMT (0.75–1.0 mm), independent of the cementation technique. A further reduction in the occlusal MMT (0.5–0.74 mm) led to a significant increase in losses caused by material fractures (HR: 11.1). The success probability of ZLS-ceramic partial crowns was significantly influenced by the tooth position, with molar restorations demonstrating a 5.26-fold increased risk for a failure or need for a clinical intervention.

Cementation of the ZLS-ceramic partial crowns with SAC showed a pronounced tendency (HR: 9.2) toward an increased rate of retention loss compared to restorations luted with a TEC material. Clinical evaluations with longer observational periods and larger sample sizes are essential for further examination of these potential influencing factors.
